# Detection of common pathogenesis of rheumatoid arthritis and atherosclerosis via microarray data analysis

**DOI:** 10.1016/j.heliyon.2024.e28029

**Published:** 2024-03-29

**Authors:** Fan Xu, Linfeng Xie, Jian He, Qiuyu Huang, Yanming Shen, Liangwan Chen, Xiaohong Zeng

**Affiliations:** a Department of Cardiovascular Surgery, Fujian Medical University Union Hospital, Fuzhou, Fujian Province, China; b Key Laboratory of Cardio-Thoracic Surgery (Fujian Medical University), Fujian Province University, Fuzhou, Fujian Province, China; c Fujian Medical University, Fuzhou, Fujian Province, China; d Department of Rheumatology, The First Affiliated Hospital of Fujian Medical University, Fuzhou, Fujian Province, China

**Keywords:** Rheumatoid arthritis, Atherosclerosis, Bioinformatics, Differentially expressed genes, Hub genes

## Abstract

Despite extensive research reveal rheumatoid arthritis (RA) is related to atherosclerosis (AS), common pathogenesis between these two diseases still needs to be explored. In current study, we explored the common pathogenesis between rheumatoid arthritis (RA) and atherosclerosis (AS) by identifying 297 Differentially Expressed Genes (DEGs) associated with both diseases. Through KEGG and GO functional analysis, we highlighted the correlation of these DEGs with crucial biological processes such as the vesicle transport, immune system process, signaling receptor binding, chemokine signaling and many others. Employing Protein-Protein Interaction (PPI) network analysis, we elucidated the associations between DEGs, revealing three gene modules enriched in immune system process, vesicle, signaling receptor binding, Pertussis, and among others. Additionally, through CytoHubba analysis, we pinpointed 11 hub genes integral to intergrin-mediated signaling pathway, plasma membrane, phosphotyrosine binding, chemokine signaling pathway and so on. Further investigation via the TRRUST database identified two key Transcription Factors (TFs), SPI1 and RELA, closely linked with these hub genes, shedding light on their regulatory roles. Finally, leveraging the collective insights from hub genes and TFs, we proposed 10 potential drug candidates targeting the molecular mechanisms underlying RA and AS pathogenesis. Further investigation on xCell revealed that 14 types of cells were all different in both AS and RA. This study underscores the shared pathogenic mechanisms, pivotal genes, and potential therapeutic interventions bridging RA and AS, offering valuable insights for future research and clinical management strategies.

## 1 Introduction

More and more evidences show that rheumatoid arthritis (RA) has a strong association with cardiovascular (CV) diseases, and the main reasons for mortality in RA patients are cardiovascular diseases and sudden death [1]. It is reported that in RA patients, due to the acceleration of atherosclerosis, the risk of cardiovascular events increases by about 30%–60%, and that of cardiovascular death increases by about 50% [2]. Recently, a meta-analysis demonstrated that RA patients exhibited increased coronary calcium scores [3]. Remarkably, RA is related with traditional CV risk factors [4], subclinical atherosclerosis [5,6], arrhythmias [7], and coronary calcifications [8,9].

Studies have proved that numerous factors contribute to cardiovascular disease in RA, including hypertension [10,11], abnormal lipid metabolism [12], obesity [13], smoking [14], and so on. In addition, there are shared molecular features between RA and AS. An inflammatory cytokine, tumor necrosis factor-alpha (TNF-α), is up-regulated in the synovial fluid of patients with RA. It plays a critical role in mediating premature atherogenesis through p38 signaling pathway, MAPK signaling pathway and NF-κB signaling pathway [15,16]. TNF-α synergizes with interleukin-6 (IL-6) forming lipid-laden macrophages (foam cells), which are crucial in atherosclerotic plaque development [[17], [18], [19], [20]]. Studies have shown that TNF-α inhibition significantly improves vascular function in RA patients [21]. Inflammatory cytokines, immune cells and many others play vital roles in both RA and the development of AS [22,23]. Latest research indicates a positive association between genetic predisposition to RA and high risk of coronary AS.

Therefore, it's essential to discover more precise biological targets in RA patients to reduce the incidence and prevent the progression of AS. In recent years, bioinformatics analysis has been considered as one of the most crucial methods in medical scientific research [24]. Through comprehensive analysis of numerous datasets and genes, bioinformatics enables the identification and assessment of pathways involved in the biological processes correlated with RA and AS. In present study, we used bioinformatics analysis methods to detect the hub genes in RA and AS. Then, we conducted enrichment analyses using Kyoto Encyclopedia of Genes and Genomes (KEGG) and Gene Ontology (GO) to elucidate the functions of these hub genes. Besides, we explored the interactions between these genes by constructing a Protein-Protein Interaction (PPI) network. Subsequently, we have verified the expression of the hub genes and their clinical prognosis. 10 possible drug molecules were also affirmed by Enrichr from the DSigDB database. Investigation of the hub genes between RA and AS could explain new perception into biological mechanisms of two diseases.

## 2 Results

### 2.1 Identification of DEGs

**Fig. 1:**
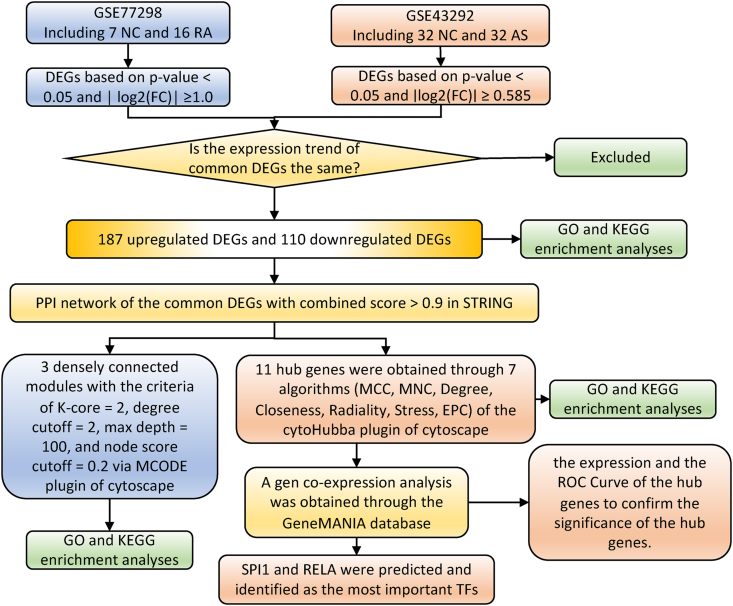
Research design flow chart.

**Fig. 2:**
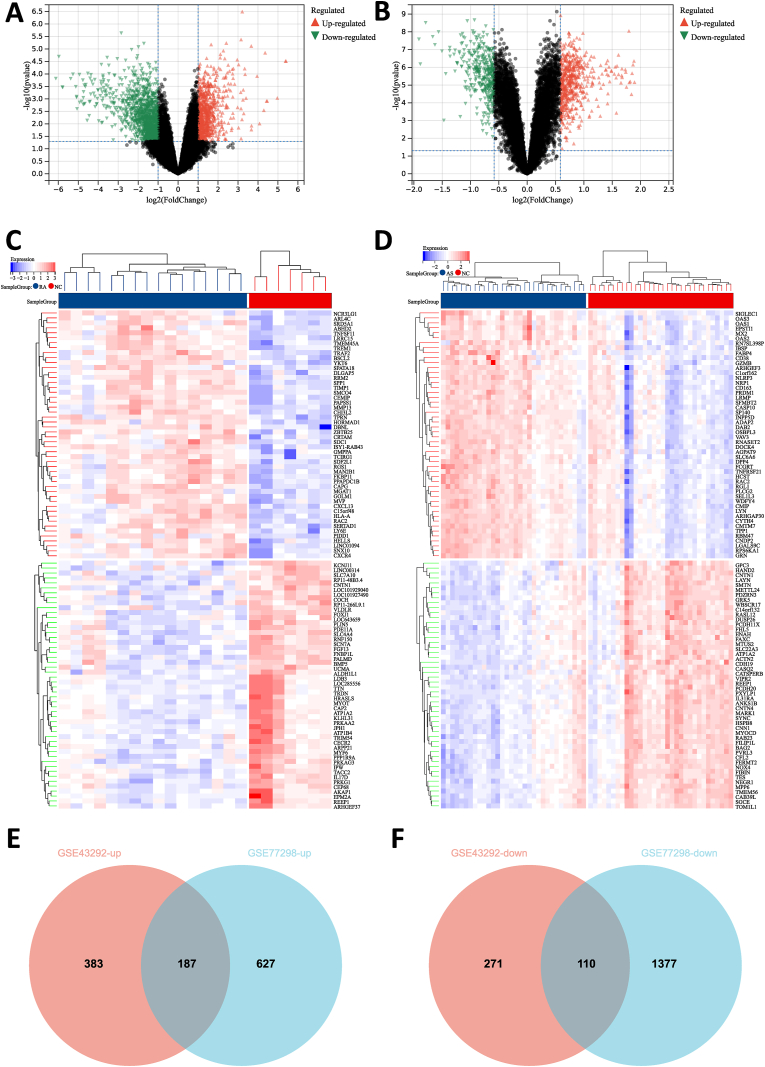
**Volcano diagram, heatmaps and Venn diagram of DEGs.** (A) The volcano map of GSE77298. (B) The volcano map of GSE43292. Upregulated genes are marked in light red; downregulated genes are marked in light green. (C) The heatmaps of GSE77298. (D) The heatmaps of GSE43292. Venn diagram of DEGs common to all 2 GEO datasets. (E) Upregulated genes. (F) Downregulated genes. (For interpretation of the references to color in this figure legend, the reader is referred to the Web version of this article.)

During present study, we structured a research flowchart that is shown in Fig. 1. After careful and conscientious screening, we obtained the gene expression profiles of GSE77298 and GSE43292 to explore the pathogenesis of rheumatoid arthritis complicated with atherosclerosis. Comparing 16 RA patients with 7 healthy controls based on criteria, which is | log2(FC)| ≥1.0, p-value <0.05, we explored 2301 DEGs from GSE77298, which include 814 up-regulated genes and 1487 down-regulated genes. And in GSE43292, there are 951 DEGs including 570 upregulated genes and 381 downregulated genes, compared 32 atherosclerotic lesions with 32 control arteries (Table 1), according to the specified cutoff criteria (∣log2(FC)∣ ≥ 0.585, p-value <0.05) (Fig. 2A and B). The top 50 DEGs are shown in the cluster heatmaps (Fig. 2C and D). In this particular study, the common DEGs were identified by dysregulated in both datasets and changed in the same direction. Therefore, the Venn analysis was applied to observe the junction of the upregulated or downregulated DEGs. Subsequently, 297 DEGs were considered as significantly differentially expressed in both groups, containing 187 conspicuously high-expressed DEGs (Figs. 2E) and 110 low-expressed DEGs (Fig. 2F).

**Table 1:** DEGs Statistics of 2 microarray databases.

Dataset ID	Up-regulated genes	Down-regulated genes	DEGs	p-value <	| log2(FC)| ≥
GSE77298	814	1487	2301	0.05	1.0
GSE43292	570	381	951	0.05	0.585

### 2.2 Investigation of the functional properties of common DEGs

**Fig. 3:**
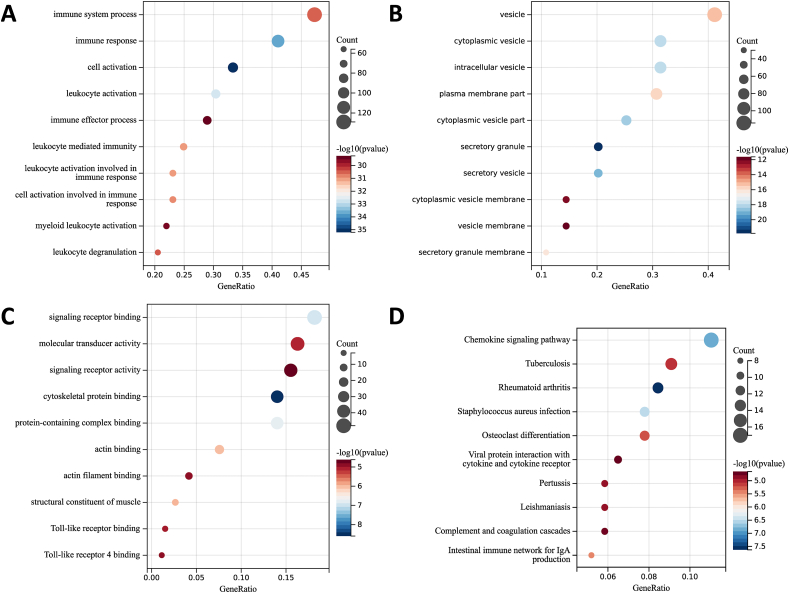
**Results of common DEGs enrichment analysis.** (A) GO biological process enrichment analysis results, (B) GO cellular component enrichment analysis results, (C) GO molecular function enrichment analysis results, (D) Results of KEGG Pathway enrichment analysis. The significant difference was considered by adjusted p-value <0.05. And the circle size represents the involved genes number, and the abscissa represents the frequency of genes, which are involved in total genes term.

The analysis of Differentially Expressed Genes (DEGs) was conducted using KEGG and GO functional enrichment analyses to explore their biological functions of DEGs in RA and AS. Understanding the biological functions of these DEGs is crucial for a better comprehension of the common pathogenesis between RA and AS. The enriched GO terms were distinguished into BP, CC and MF ontologies. Significant terms were obtained according to p-value <0.05, with top 10 terms deemed as significant. In GO analysis, the result showed that DEGs were correlated with various biological processes including immune system process, cell activation, immune response, immune effector process, leukocyte activation, leukocyte mediated immunity, leukocyte activation involved in immune response, cell activation involved in immune response, myeloid leukocyte activation and leukocyte degranulation (Fig. 3A). Regarding cellular components, DEGs were enriched mainly in secretory granule, secretory vesicle, cytoplasmic vesicle part, cytoplasmic vesicle, intracellular vesicle, secretory granule membrane, plasma membrane part, vesicle, cytoplasmic vesicle membrane and vesicle membrane (Fig. 3B). MF analysis demonstrated abundant enrichment of DEGs in cytoskeletal protein binding, signaling receptor binding, protein-containing complex binding, actin binding, structural constituent of muscle, molecular transducer activity, Toll-like receptor binding, Toll-like receptor 4 binding, actin filament binding and signaling receptor activity (Fig. 3C). Moreover, KEGG pathway analysis results highlighted that DEGs were predominantly enriched in Rheumatoid arthritis, Chemokine signaling pathway, Tuberculosis, *Staphylococcus aureus* infection, Intestinal immune network for IgA production, Osteoclast differentiation, Complement and coagulation cascades, Pertussis, Leishmaniasis, and Viral protein interaction with cytokine and cytokine receptor (Fig. 3D). Utilizing ClueGo for functional analysis corroborated these findings. Overall, these results provide valuable insights into the biological functions of DEGs and their potential roles in the pathogenesis of RA and AS.

### 2.3 PPI network construction and module analysis

**Fig. 4:**
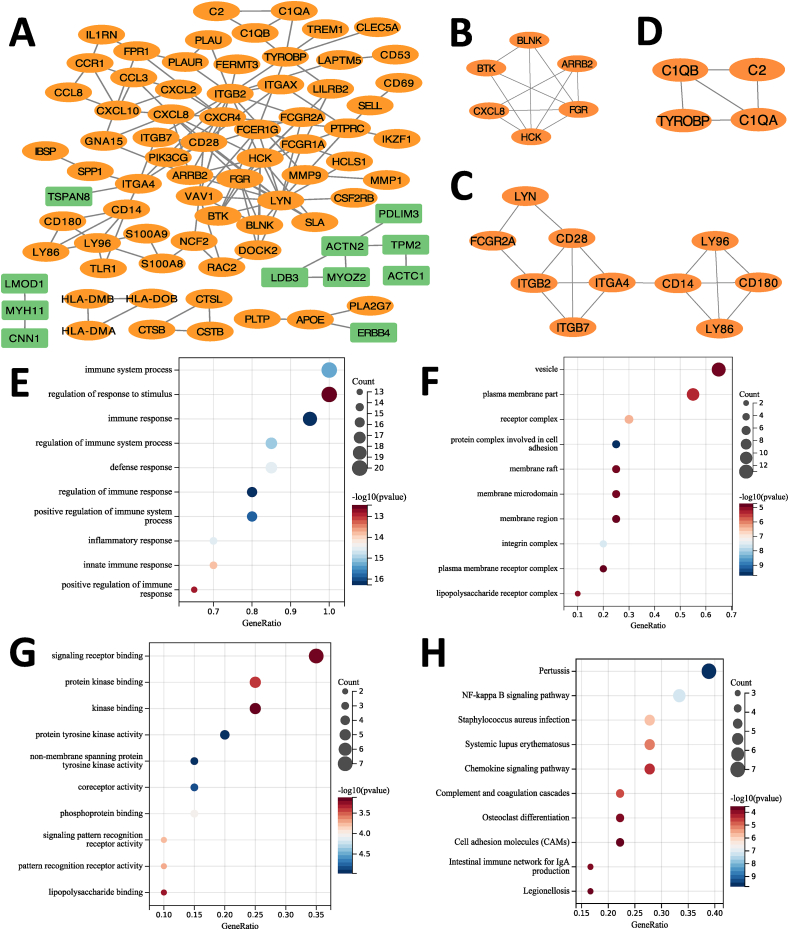
**PPI network, significant gene modules and enrichment analysis results of the modular genes.** (A) Diagram of PPI network. Orange shows up-regulated genes, and green shows down-regulated genes. (B, C, D) Three significant gene clustering modules. (E) The enrichment analysis results of GO BP, (F) The enrichment analysis results of GO CC, (G) The enrichment analysis results of GO MF, (H) Results of KEGG Pathway enrichment analysis. Adjusted p-value <0.05 was considered as significantly different. The circle size mentions the involved gene numbers, and the abscissa proves the frequency of the genes contained in the term total genes. (For interpretation of the references to color in this figure legend, the reader is referred to the Web version of this article.)

To gain deeper insights into the cellular functions and molecular mechanisms underlying RA and AS, we utilized Protein-Protein Interaction (PPI) network analysis. This involved utilizing the STRING online database and Cytoscape to construct the PPI network, which comprised 79 nodes and 138 interaction pairs (Fig. 4A). Highly expressed genes were shown in orange color while the low expressed genes were represented in green. Subsequently, MCODE, a Cytoscape plugin, was applied to identify three closely connected gene modules, which include 20 common DEGs and 32 interaction pairs (Fig. 4B–D). GO and KEGG functional enrichment analyses were processed for these common DEGs. As the results shown in the GO analysis, BP enrichment analysis revealed that these common DEGs were correlated with immune system process, regulation of response to stimulus, immune response, etc. (Fig. 4E). In terms of CC enrichment analysis, the common DEGs were abundant mostly in vesicle, plasma membrane part, receptor complex, and so on (Fig. 4F). MF analysis indicated that the common DEGs were principally enriched in signaling receptor binding, protein kinase binding, kinase binding, etc. (Fig. 4G). Furthermore, KEGG enrichment analyses showed the common DEGs were enriched in Pertussis, NF-kappa B signaling pathway, *Staphylococcus aureus* infection, and so on (Fig. 4H).

### 2.4 Selection and analysis of hub genes

CytoHubba was applied to select the hub genes by calculating top 20 hub genes through 7 algorithms, including MCC, MNC, Degree, Closeness, Radiality, Stress and EPC (Table 2).

**Table 2:** The list of the top 20 hub genes in cytoHubba.

MCC	MNC	Degree	Closeness	Radiality	Stress	EPC
LYN	LYN	LYN	ITGB2	ITGB2	ITGB2	HCK
HCK	HCK	ITGB2	LYN	LYN	LYN	LYN
FGR	FGR	HCK	HCK	HCK	CXCL8	FGR
CXCL8	CD28	FGR	VAV1	VAV1	ITGA4	FCER1G
ARRB2	ARRB2	CXCL8	CD28	CD28	HCK	FCGR2A
BTK	CXCL8	VAV1	FCER1G	FCGR2A	VAV1	ITGB2
BLNK	FCER1G	FCER1G	FGR	FCER1G	TYROBP	CXCL8
ITGB2	ITGB2	CXCR4	FCGR2A	ITGA4	CXCR4	VAV1
CD28	FCGR2A	TYROBP	CXCL8	TYROBP	PTPRC	ARRB2
FCER1G	BLNK	LY96	TYROBP	CXCL8	FCER1G	BLNK
VAV1	VAV1	ITGA4	CXCR4	CXCR4	CD14	BTK
FCGR2A	BTK	CD28	ITGA4	FGR	CD28	CXCR4
LY96	CXCL10	PTPRC	PTPRC	PTPRC	CXCL10	FCGR1A
ITGA4	LY96	FCGR2A	ARRB2	ARRB2	NCF2	CD28
CD14	FCGR1A	ARRB2	BLNK	ITGAX	FPR1	PTPRC
FCGR1A	CCL3	FCGR1A	FCGR1A	ITGB7	FCGR2A	DOCK2
CXCL10	CD14	BLNK	BTK	PLAUR	ARRB2	SLA
TYROBP	ITGA4	BTK	ITGAX	BLNK	FGR	TYROBP
CXCR4	LY86	CXCL10	DOCK2	FCGR1A	S100A8	ITGA4
DOCK2	ITGB7	CD14	ITGB7	BTK	CCL3	ITGAX

**Fig. 5:**
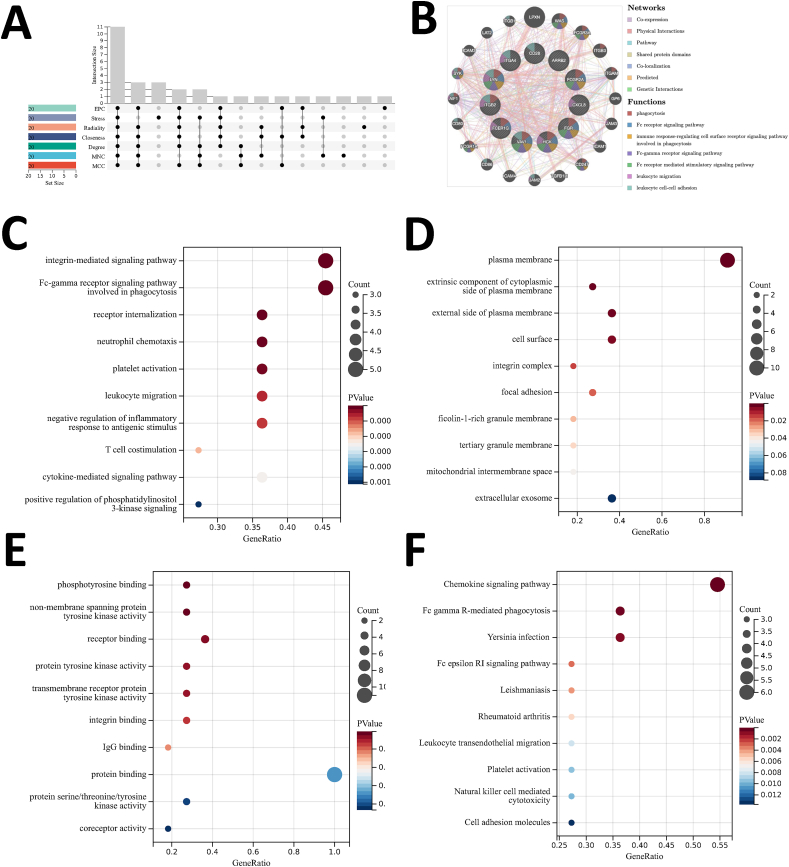
**Venn diagram and co-expression network of hub genes.** (A) The Venn diagram indicated that seven algorithms have extracted 11 overlapping hub genes. (B) GeneMANIA analysis of hub genes and co-expression genes. (C) GO BP enrichment analysis results (D) The enrichment analysis results of GO CC, (E) GO MF enrichment analysis results, (F) KEGG Pathway enrichment analysis results. Adjusted p-value <0.05 was regarded as significantly different. The circle size explains involved genes number, and the abscissa shows frequency of the genes that are included in the term total genes.

Then, we used Venn diagrams to obtain the common hub genes, revealing 11 significant genes shared between RA and AS, namely LYN, HCK, FGR, CXCL8, ARRB2, ITGB2, CD28, FCER1G, VAV1, FCGR2A and ITGA4 (Fig. 5A). We also used GeneMANIA online database analyzing the co-expression network and related functions of these hub genes. The result proved that those 11 DEGs exhibited the delicate DEGs PPI network formed by Co-expression of 62.63%, Physical Interactions of 25.96%, Pathway of 4.58%, Shared protein domains of 2.59%, Co-localization of 2.39%, Predicted of 1.53%, and Genetic interactions of 0.33% (Fig. 5B). GO analysis unveiled that hub genes were mostly associated with various biological processes (BP) including integrin-mediated signaling pathway, receptor internalization, Fc-gamma receptor signaling pathway involved in phagocytosis, neutrophil chemotaxis, platelet activation, leukocyte migration, negative regulation of inflammatory response to antigenic stimulus, T-cell co-stimulation, cytokine-mediated signaling pathway and positive regulation of phosphatidylinositol 3-kinase signaling (Fig. 5C). Concerning cellular components, the hub genes were enriched in plasma membrane, extrinsic component of cytoplasmic side of plasma membrane, external side of plasma membrane, cell surface, integrin complex, focal adhesion, ficolin-1-rich granule membrane, tertiary granule membrane, mitochondrial intermembrane space and extracellular exosome (Fig. 5D). MF analysis presented that the hub genes were mostly enriched in phosphotyrosine binding, non-membrane spanning protein tyrosine kinase activity, receptor binding, protein tyrosine kinase activity, transmembrane receptor protein tyrosine kinase activity, integrin binding, IgG binding, protein binding, protein serine/threonine/tyrosine kinase activity and coreceptor activity (Fig. 5E). Additionally, KEGG pathway analysis results revealed that the hub genes were primarily enriched in Fc gamma R-mediated phagocytosis, Chemokine signaling pathway, Yersinia infection, Fc epsilon RI signaling pathway, Leishmaniasis, Rheumatoid arthritis, Leukocyte *trans*-endothelial migration, Platelet activation, Natural killer cell mediated cytotoxicity and Cell adhesion molecules (Fig. 5F).

### 2.5 ROC curve and validation of hub genes

**Fig. 6:**
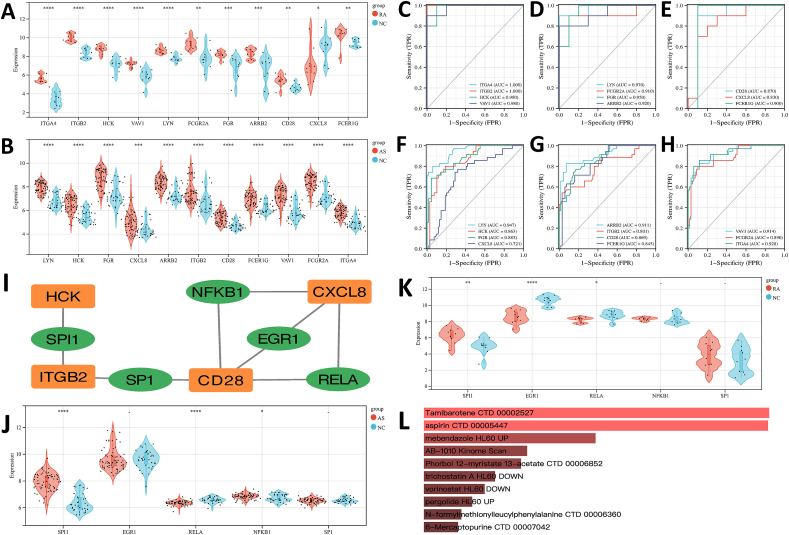
**The expression level and ROC Curve of hub gene in GSE55235 and GSE100927.** (A) The expression level in GSE55235. (B) The expression level in GSE100927. The mean T test was applied to make comparison between the two sets of data. The statistical significance was considered by adjusted p*-*value <0.05. *P < 0.05; ***P < 0.001; ****P < 0.0001. (C) ROC curve of ITGA4, ITGB2, HCK and VAV1 in GSE55235. (D) ROC curve of LYN, FCGR2A, FGR and ARRB2 in GSE55235. (E) ROC curve of CD28, CXCL8 and FCER1G in GSE55235. (F) ROC curve of LYN, HCK, FGR and CXCL8 in GSE100927. (G) ROC curve of ARRB2, ITGB2, CD28 and FCER1G in GSE100927. (H) ROC curve of VAV1, FCGR2A and ITGA4 in GSE100927. AUC area under the ROC curve. (I) Transcription Factor regulatory network. TFs were demonstrated in green and hub genes were demonstrated in orange. (J, K) The level of TFs expression in GSE55235 and GSE100927. The mean T-test was used for comparing the two data sets. Significant difference was evaluated by p*-*value <0.05. *P < 0.05; **P < 0.01; ****P < 0.0001. (L) Identification of 10 candidate drugs. (For interpretation of the references to color in this figure legend, the reader is referred to the Web version of this article.)

The validation of 11 hub genes was done by using two other datasets, GSE55235 and GSE100927, to ensure the reliability of their expression levels. The result demonstrates that compared 10 RA joints with 10 healthy joints in the GSE55235 dataset, the selected hub genes were conspicuously up-regulated in the RA patients, except for CXCL8, which was down-regulated in the RA patients (Fig. 6A). Furthermore, in the GSE100927 dataset consisting of 69 atherosclerotic lesions and 35 control arteries, the expression of all selected hub genes in atherosclerotic plaques was significantly up-regulated than in normal tissues (Fig. 6B). Then, we analyzed the prognostic value of the hub genes to assess their reliability. These hub genes exhibited greater diagnostic value in the patients’ samples when compared to normal samples. All of the hub genes have a high diagnostic value (Fig. 6C–H). Due to their good diagnostic performance, we hypothesize that the 11 hub genes could serve as biomarkers for the diagnosis of both RA and AS.

### 2.6 Prediction and validation of transcription factors

The TRRUST database was utilized to identify key transcription factors (TFs), which regulate the expression of hub genes. In this study, 5 TFs were identified as pivotal regulators: SP1, SPI1, NFKB1, EGR1 and RELA (Fig. 6I).

Further verification, based on GSE55235 and GSE100927, we found SPI1, EGR1 and RELA were significantly associated with RA patients (Fig. 6J). Meanwhile, SPI1, RELA and NFKB1 were considered as statistically significant in AS patients (Fig. 6K). Finally, SPI1 and RELA emerged as the most crucial TFs, with SPI1 showing upregulation in both diseases, whereas RELA exhibited downregulation. These findings underscore the regulating action of SPI1 and RELA in the pathogenesis of RA and AS.

### 2.7 Identification of candidate drugs

To better understand the significance of hub genes and explore potential therapeutic options for diseases, we evaluated the protein-drug interactions. Through Enrichr, leveraging transcriptome signatures from the DSigDB database, we identified 10 possible drug molecules based on their adjusted P-values (Fig. 6L). The effective drugs targeting hub DEGs from the DSigDB database were further detailed in Table 3. These findings provide valuable information for potential therapeutic interventions targeting the hub genes associated with RA and AS.

**Table 3:** List of the 10 candidate drugs.

Name	Adjusted P-value	Chemical Formula	Structure
Tamibarotene CTD 00002527	2.21E-07	C22H25NO3	
aspirin CTD 00005447	2.21E-07	C9H8O4	
mebendazole HL60 UP	1.41E-05	C16H13N3O3	
AB-1010 Kinome Scan	6.10E-05	C28H30N6OS	
Phorbol 12-myristate 13-acetate CTD 00006852	6.10E-05	C36H56O8	
trichostatin A HL60 DOWN	1.01E-04	C17H22N2O3	
vorinostat HL60 DOWN	1.14E-04	C14H20N2O3	
pergolide HL60 UP	1.39E-04	C19H26N2S	
N-formylmethionylleucylphenylalanine CTD 00006360	1.41E-04	C21H31N3O5S	
6-Mercaptopurine CTD 00007042	1.41E-04	C5H4N4S	

### 2.8 Role of immune cell infiltration in RA and AS

**Fig. 7:**
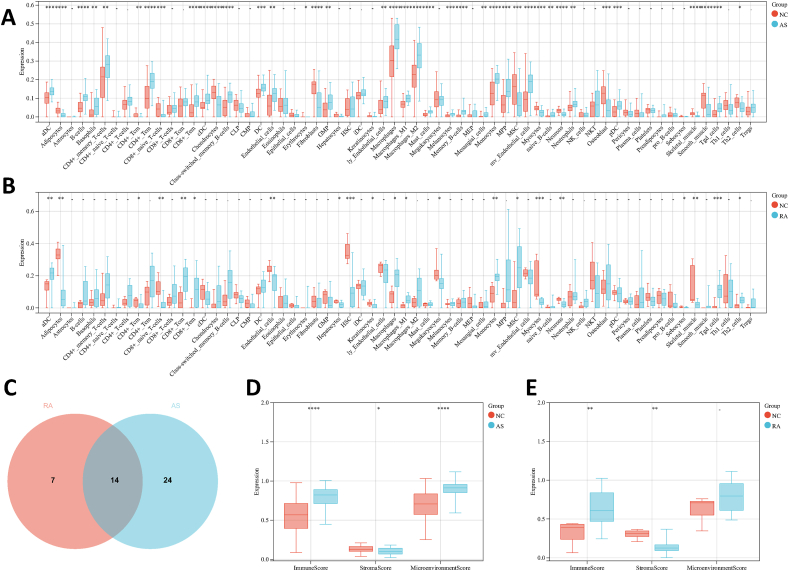
**Immune cell infiltrates between disease and control groups in AS and RA.** (A) Immune cell infiltrates in AS. (B) Immune cell infiltrates in RA. (C) Common immune cell infiltrates in RA and AS. (D) ImmuneScore, StromaScore, and MicroenvironmentScore in RA. (E) ImmuneScore, StromaScore, and MicroenvironmentScore in AS.

xCell was used to find out the differences about the infiltration of immune cell between disease and control groups in AS and RA. Myocytes, HSC, Tgd_cells, Monocytes, Skeletal_muscle, and so on, have significant differences between RA and NC (Fig. 7A). Similarly, we also find that Macrophages_M1, Skeletal_muscle, Mast_cells, Myocytes, B-cells, and so on, were distributed differently in AS and NC (Fig. 7B). Further investigation revealed that 14 types of cells, including Myocytes, Tgd_cells, Monocytes, Skeletal_muscle, Adipocytes, CD8+_naive_T-cells, Endothelial_cells, aDC, CD8+_Tem, CD4+_Tcm, Macrophages, MSC, Macrophages_M1 and Th2_cells, were all different in AS and RA. (Fig. 7C). Interestingly, in AS, Endothelial_cells were highly and MSC and Th2_cells were low contrasted with NC. But in RA, these are precisely the opposite. At the same time, we also analyzed the differences in ImmuneScore, StromaScore, and MicroenvironmentScore between disease group and control group in two types of diseases. The result show that ImmuneScore was significantly high in the disease group in two types of diseases. (Fig. 7D and E).

## 3 Discussion

Rheumatoid arthritis (RA) is a chronic, systemic inflammatory disease, which might be progressive with unclear etiology and it has induced local (joint) and systemic inflammatory manifestations approximately 0.2–1% of adults worldwide for long term [[25], [26], [27]]. In addition, atherosclerosis is an inflammatory condition. Chronic and systemic inflammation can cause AS(28) and contribute to cognitive impairment [28]. More seriously, as an autoimmune inflammatory disease, RA can lead to cardiovascular diseases, including AS, cerebrovascular diseases, peripheral vascular diseases and heart failure [29,30]. A study reported that RA patients had a higher risk in progression into the cardiovascular diseases (CVD) than normal population [31]. There are studies demonstrating that RA and AS have common pathogenesis. Choy E. H et al. have proved that inflammation can cause various diseases, such as rheumatoid arthritis [32], cardiovascular diseases [33], and even cancer [34]. Rattazzi M et al. deemed atherosclerosis [35,36] and rheumatoid arthritis [37] to be correlated with interleukin-6. Our study aims to comprehensively explore the common key pathogenic genes and related mechanisms of RA and AS through bioinformatics analysis of microarray data. This research lays the foundation for subsequent studies in this field.

To date, there are only a few studies which use advanced bioinformatics methods in exploring the common molecular mechanism among these two diseases. Microarray technology is an effective analytical tool widely utilized for comparing DEGs in various biological models or patients across different disease states [38,39]. The application of weighted gene co-expression network analysis (WGCNA) by Zhang et al. in identifying key modules and hub genes in oral squamous cell carcinoma (OSCC) tumorigenesis has significantly advanced research in the field of oral cancer [40].

In this study, four datasets were used to explore the co-pathogenesis of RA and AS. Using comprehensive bioinformatics analysis, we have identified 297 DEGs as significant DEGs in both groups, including 187 significantly up-regulated DEGs and 110 down-regulated DEGs. GO analysis revealed that DEGs were related with immunes such as immune response, immune system process, cell activation, and so on. KEGG analysis identified that DEGs were abundant in Rheumatoid arthritis, Chemokine signaling pathway, and so on. Then, we applied PPI network and MCODE to understand the molecular mechanisms better. The GO and KEGG analysis provided that these DEGs from the three gene modules were also associated with immune.

In current study, via seven algorithms in CytoHubba, we meticulously selected 11 hub genes and 2 important TFs associated with the pathogenesis of RA and AS. Through functional analysis, we elucidated their roles in the respective diseases. Notably, Hu et al. proved that Dihydroarteannuin was found to ameliorate collagen-induced arthritis by inhibiting B cell activation through the FcγRIIb/Lyn/SHP-1 pathway [41]. Additionally, You et al. investigated that sorting Nexin 10 mediated metabolic reprogramming of macrophages in atherosclerosis via the Lyn-dependent TFEB signaling pathway [42]. Medina et al. showed that deficiency of Hck/Fgr kinase reduced plaque growth and stability by receding monocyte recruitment and intraplaque motility [43], although its role in RA remains unexplored. Furthermore, CXCL8, IL-8, Antcin K, and ARRB2 were implicated in regulating inflammation and cartilage degradation in RA(45), while neutrophil extracellular traps aggravated AS in macrophages via NF-κB signaling [44,45].

Tian et al. demonstrated that lncRNA AC078850.1 induced NLRP3 inflammasome-mediated pyroptosis in AS by increasing ITGB2 transcription by means of HIF-1α [46]. Genetic polymorphisms in CTLA-4, CD80/86, CD28, and FCER1G were correlated with RA pathogenesis, including gene hypomethylation in FCER1G [47]. Moreover, VAV1 regulation was linked to experimental autoimmune arthritis and anti-CCP negative RA [48], while FCGR2A variation was associated with the response to anti-TNF therapy in RA and the presence of atherosclerotic plaques in carotid arteries [[49], [50], [51]]. Additionally, the miR-30s family was found to modulate proliferation and apoptosis in human coronary artery endothelial cells by targeting ITGA4 and PLCG1(54). These findings highlight the intricate molecular mechanisms underlying RA and AS, providing insights into potential therapeutic targets for these diseases.

We also tried our best to find the candidate drugs and we used Enrichr to choose 10 possible drug molecules depending on their adjusted P-value. A study proved that dopamine used the DRD5-ARRB2-PP2A Signaling Axis blocking TRAF6-Mediated NF-κB Pathway and suppressing the systemic inflammation [52]. Katzav S et al. described Vav1 as an oncogene which manages the specific transcriptional activation of T cells [53]. Studies have shown that iTGB2(57), CD28(58), FCER1G(59), FCGR2A(60), ITGA4(61) are associated with a variety of diseases and participate in immune response. Studies have shown that synthetic retinoid tamibarotene can inhibit T follicular helper and Th17 cell responses to ameliorate arthritic autoimmune responses [54] and reduce atherosclerosis in mice by inhibiting IL-6(63). A study indicated that treatments with retinoids were effective for arthritis, nephritis, myositis, and vasculitis in experimental animal models because retinoids have immunoregulatory functions [55]. However, there is still no report about this drug treating AS to date.

Aspirin has played an important role in cardiovascular disease [56] and rheumatoid arthritis therapy [57] due to its anti-inflammatory and anti-platelet effects. Mebendazole, methyl 5-benzoyl-1H-benzimidazol-2-yl-carbamate, is a broad-spectrum anthelmintic, but it also has anti-cancer effects on several kinds of solid tumors, such as ovarian cancer [58], pancreatic cancer [59], gliomas [60] etc. Phorbol 12-myristate 13-acetate (PMA) is a potential promoter of cancer and very inflammatory in nature. However, it can induce cardiac fibrosis [61], rodent brain in rats [62] and can induce toxicity study [63,64]. PMA-induced differentiation of monocytic THP-1 cells into macrophages in atherosclerosis [65] and induced oxyradical production in rheumatoid synovial cells [66]. Trichostatin A can worsen AS in low-density lipoprotein receptor-deficient mice [67] and inhibit RA fibroblast-like synoviocyte and macrophage IL-6 production by expediting mRNA decay [68]. It can inhibit the proliferation of vascular smooth muscle cells, which can lead to AS and post-angioplasty restenosis, via induction of p21(WAF1) [69].

SAHA (vorinostat) exhibited their anti-rheumatic activities by growth arrest in RA synovial fibroblasts, inhibition of pro-inflammatory cytokines and NO, as well as down-regulation in angiogenesis and MMPs [70]. Moreover, SAHA can repress vascular inflammation and AS(80). Pergolide is an oral dopamine receptor agonist, which is broadly used in treating Parkinson disease [71]. Topical Pergolide can promote NGF upregulation inducing corneal nerve regrowth and thus promote corneal sensation [72]. N-formyl-methionyl-leucyl-phenylalanine (fMLP) is potentially chemo-attractractive for neutrophils, which was utilized in periprosthetic joint infection mouse model with *S. aureus*. fMLP treatment group revealed relieve in pain and increased weight-bearing at the implant leg of infected mice [73].

A study emphasized on evaluating the [3H]PK11195 binding parameters in acute inflammation model, the fMLP-stimulated neutrophil cell membranes, and analyzing if alterations of peripheral-type benzodiazepine receptor (PBR) characteristics occurred in neutrophil cell membranes of patients affected by osteoarthritis (OA), RA and psoriasic arthritis (PA). The results suggested that there was a fMLP modulation on [3H]PK11195 binding in human neutrophils [74]. 6-mercaptopurine inhibits AS in apolipoprotein e*3-leiden transgenic mice via atheroprotective actions on monocytes and macrophages [75] and is associated with RA [76]. Masitinib also is related to Rheumatoid arthritis [77], while there are not studies to research the relationship between Mebendazole and AS or RA.

Over the past decade, research has revealed that RA and AS share similar pathogenesis and common risk factors [78,79], with chronic inflammation and immune activation emerging as pivotal factors [80,81]. Activation of both innate and adaptive immune systems contributes to elevated cumulative inflammation, involving the production of cytokines such as TNF-α and IL-6, activation of T and B cells, and an increase in various non-immune cells including fibroblasts, epithelial cells, and smooth muscle cells [78,82,83]. Furthermore, chronic inflammation both exacerbates traditional risk factors and alternates with the underlying mechanisms [78]. This complex biological process generates endothelial dysfunction, arterial stiffness, and the formation and progression of atherosclerotic plaques, ultimately accelerating the atherogenic process in RA [79,80,84,85]. In our study, the infiltration of immune cells was observed in RA and AS, which also indirectly reflects the relevance of Myocytes, Tgd_cells, Monocytes, Skeletal_muscle, Adipocytes, CD8+_naive_T-cells, Endothelial_cells, aDC, CD8+_Tem, CD4+_Tcm, Macrophages, MSC, Macrophages_M1 and Th2_cells to AS and RA.

Our study also has several limitations. Firstly, we relied on public databases, and the limited number of samples within the datasets may introduce some uncertainty in the data analysis. Secondly, although we validated our results using additional datasets, further validation through *in vivo* and *in vitro* experiments is necessary. Thirdly, the clinical prognostic values were analyzed using ROC curves based on a relatively small sample size, which may become more reliable with a larger sample size. Fourthly, the functionality of the drugs and immune cells we screened needs validation and further exploration in cells and animal models. Our future work will be focusing on these experiments. Additionally, we hope that the genes we have identified can inspire other researchers in the field and serve as research targets. The drugs we have screened may also advance both basic research and clinical progress in this field.

## 4 Conclusions

In this study, we have verified common pathogenic mechanisms, pathogenic genes, and potential therapeutic drugs shared between RA and AS. Our findings pointed out the common pathogenesis of RA and AS, offering insights into the potential molecular mechanisms about RA in the presence of AS. This research provides valuable implications for understanding the interconnectedness of these diseases and offers potential directions for further exploration into their molecular mechanisms.

## 5 Materials and methods

### 5.1 Dataset collection of RA and AS

GEO (http://www.ncbi.nlm.nih.gov/geo) [86]is a public repository containing enormous amount of high-throughput sequencing and microarray datasets collected by global research institutes. For our study, we selected four gene expression microarray datasets (GSE77298(97), GSE43292(98), GSE55235(99) and GSE100927(100)) from the GEO database. As experimental groups, the GSE77298 dataset, available on GPL570 platform (HG-U133_Plus_2; Affymetrix Human Genome U133 Plus 2.0 Array), comprised 7 healthy controls and 16 RA patients. The GSE43292 dataset, accessible on the GPL6244 platform [HuGene-1_0-st; Affymetrix Human Gene 1.0 ST Array], involved 32 pairs of AS lesions and control arteries. As test groups, the GSE100927 dataset was downloaded from GPL17077 platform (Agilent-039494 SurePrint G3 Human GE v2 8 × 60K Microarray 039381), which included 69 AS lesions and 35 control arteries. In addition, gene expression data in synovial tissue samples from 10 healthy joints and 10 RA joints were contained in the analysis from the GSE55235 dataset. These data were obtained using the GPL96 [HG-U133A] Affymetrix Human Genome U133A Array.

### 5.2 Identification of DEGs

Bioinformatic analysis was conducted to identify the differentially expressed genes (DEGs) in comparing the groups by Bioconductor package “limma” in R program [87,88]. DEGs in the GSE77298 dataset were determined based on the selection criteria of p-value <0.05 and |log2 (fold change (FC))| ≥1.0, For the GSE43292 dataset, DEGs were identified with a cutoff criteria of ∣log2(FC)∣ ≥ 0.585, p-value <0.05. Heatmaps and volcano plots of the DEGs were then constructed by ggplot2 R package (https://ggplot2.tidyverse.org) and the differential expression of DEGs was investigated by pheatmap R package (https://CRAN.R-project.org/package = pheatmap). These DEGs dysregulated in both GSE77298 and GSE43292 datasets, and altered in the same way across all data sets (i.e., either upregulated or downregulated together), were defined as candidate cross-talk genes linking atherosclerosis and rheumatoid arthritis. Eventually, the Venn diagram was generated to calculate the intersecting parts of DEGs using a specialized web tool [89].

### 5.3 GO and KEGG analysis

GO is a giant database founded by the Gene Ontology Federation [90]. Serving as a universal tool, it can be used to define the biological process (BP), cellular component (CC), and molecular function (MF) of enormous genes. On the other hand, KEGG is a database, which saves information such as biological pathways, genomes, chemical substances, drugs and diseases, which is widely used to ensure functional and metabolic pathways [91]. To further investigate the relationship between functions or pathways, the significant roles of DEGs were analyzed by GO and pathway analysis. We performed DEGs enrichment analyses by clusterProfiler (version 4.0) [92], and p-value<0.05 was considered as significant statistically. Moreover, ClueGo, a Cytoscape plug-in software, was employed to construct the GO and KEGG network and visualize the results.

### 5.4 Construction of PPI network and module analysis

PPI network elucidates the intricate interactions between proteins, highlighting the core protein genes. To delve into these relationships, we utilized the Search Tool for the Retrieval of Interacting Genes (STRING; http://string-db.org) [93], a freely accessible database. An interaction score of at least 0.9 (highest confidence score) was considered as significant. Then, the Cytoscape software (http://www.cytoscape.org) (version 3.7.2) [94] was employed to visualize and analyze this PPI network. In addition, molecular complex detection (MCODE), one of the Cytoscape plugins, was applied to identify and refine the gene clusters within the PPI network [95]. We utilized specific criteria, including K-core = 2, degree cutoff = 2, max depth = 100, and node score cutoff = 0.2, to select the first three parts. Finally, we comprehensively examined the involvement of modular genes through KEGG and GO analysis to gain deeper insights into their functional roles.

### 5.5 Hub genes selection and analysis and validation of expression in other data sets

The “cytoHubba” plugin of Cytoscape was preferred to identify the hub genes. Here, seven diverse algorithms (Degree, MCC, MNC, Stress, Closeness, Radiality and EPC) were applied to assess and identify hub genes. Meanwhile, we selected co-expression genes throughout UpSetR. The mRNA expression of identified hub genes was examined in GSE55235 and GSE100927. Student T-test was applied to analyze the comparison between two datasets, p-value <0.05 was considered as statistically significant. ROC curve was further used to analyze the prognostic value of the hub genes via the ‘pROC’ R package and ‘ggplot2’ R package. The area under the curve (AUC), an index combination of sensitivity and specificity, was used to evaluate the intrinsic efficacy of diagnostic tests [96]. Typically, researchers interpret AUC values as follows: AUC = 0.5 indicates no discrimination; 0.5 < AUC <0.7 indicates poor discrimination; 0.7 ≤ AUC <0.8 indicates acceptable discrimination; 0.8 ≤ AUC <0.9 indicates excellent discrimination; AUC ≥0.9 indicates outstanding discrimination.

### 5.6 Transcription factors (TFs) prediction and verification

The predictive tool, Transcriptional Regulatory Relationships Unraveled by Sentence-based Text mining (TRRUST; https://www.grnpedia.org/trrust/) [97] was applied to predict the transcriptional regulatory networks. The TRRUST database was utilized to acquire TFs which regulate hub genes and adjusted p-value <0.05 was regarded as statistical significance. Eventually, we evaluated these TFs expression in GSE55235 and GSE100927 via the T-test.

### 5.7 Evaluation of applicant drugs

One of the most crucial aspects of disease research is predicting protein-drug interaction (PDI) or identifying drug molecules. Enrichr is a finalized gene set enrichment analysis web server with extensive gene-set libraries and it can apply to explore gene-set enrichment [98]. DSigDB, a drug signatures database for gene set analysis [99], has 22,527 gene sets. In this study, the Drug Signatures database (DSigDB) was used to identify the drug molecule, via Enrichr according to the hub genes and hub TFs.

### 5.8 Estimation of immune cell infiltrations in AS and RA

The xCell algorithm is a robust tool capable of analyzing the infiltration of 64 stroma cell and immune cell types, including extracellular matrix cells, hematopoietic progenitors, epithelial cells, innate and adaptive immune cells [100]. In this study, we employed the xCell algorithm to analyze immune cell infiltrations between disease and control groups in both AS and RA. The results were carried out by using the ggplot2 package in R.

### 5.9 Statistical analysis

Statistical analysis was conducted using RStudio (https://www.R-project.org). For non-normally distributed data, the Wilcoxon rank-sum test was employed as the statistical method, while for normally distributed data, the student T-test was applied. Significance was determined at a p-value <0.05.

## Authorship confirmation/contribution statement

Xiaohong Zeng and Qiuyu Huang contributed equally to this work. Xiaohong Zeng and Qiuyu Huang collected data, analyzed the data and wrote the article. Linfeng Xie, Yanming Shen, Jian He and Hao Zhou helped to collect data and deal with the figures. Fan Xu had designed the project and provided guidance.

## Funding

The project was supported by: Joint Funds for the innovation of Science and Technology, Fujian province, China (grant number: 2018Y9047); Natural Science Foundation of Fujian Province, China (grant number: 2020J011032, 2022J01696); Fujian Provincial health technology project, China (grant number: 2019-1-29) and Key Laboratory of Cardio-Thoracic Surgery (Fujian Medical University), Fujian Province University Construction Project (grant number: 2019–67).

## Data availability statement

All data to support the conclusions have been either provided or are otherwise publicly available.

## CRediT authorship contribution statement

**Fan Xu:** Writing – original draft, Validation, Software, Methodology, Investigation, Formal analysis, Data curation, Conceptualization. **Linfeng Xie:** Writing – original draft, Validation, Software, Investigation, Formal analysis, Data curation. **Jian He:** Validation, Software, Investigation, Data curation. **Qiuyu Huang:** Investigation, Formal analysis, Data curation. **Yanming Shen:** Validation, Software, Formal analysis. **Liangwan Chen:** Writing – review & editing, Supervision, Resources, Funding acquisition, Conceptualization. **Xiaohong Zeng:** Writing – review & editing, Visualization, Supervision, Resources, Project administration, Funding acquisition, Conceptualization.

## Declaration of competing interest

The authors declare the following financial interests/personal relationships which may be considered as potential competing interests: Fan Xu reports financial support was provided by Joint Funds for the innovation of Science and Technology. Fan Xu reports financial support was provided by Natural Science Foundation of Fujian Province. Fan Xu reports financial support was provided by Fujian Provincial health technology project. Xiaohong Zeng reports financial support was provided by Natural Science Foundation of Fujian Province. Liangwan Chen reports financial support was provided by Fujian Province University Construction Project. Liangwan Chen reports financial support was provided by Key Laboratory of Cardio-Thoracic Surgery. If there are other authors, they declare that they have no known competing financial interests or personal relationships that could have appeared to influence the work reported in this paper.
